# Primary Malignant Fibrous Histiocytoma of the Lung: A Case Report

**DOI:** 10.1155/2010/389692

**Published:** 2010-07-05

**Authors:** I. Tsangaridou, G. Papamihalis, K. Stathopoulos, O. Konstantinopoulos, L. Thanos

**Affiliations:** ^1^Department of Medical Imaging and Interventional Radiology, “Sotiria” Athens General Hospital of Chest Diseases, Greece; ^2^First Department of Thoracic Surgery, “Sotiria” Athens General Hospital of Chest Diseases, 11527 Athens, Greece

## Abstract

Primary malignant fibrous histiocytoma (MFH) of the lung is extremely rare although it is among the most common soft tissue sarcomas in adults. Surgery is the primary mode of therapy, with high rates of local and distant recurrence, while radiation therapy appears to be a very useful adjunct, decreasing local relapse. We report a case of primary malignant fibrous histiocytoma of the lung. Fourteen years after surgical resection, the patient is still alive although with multiple metastatic lesions throughout his body.

## 1. Introduction

Malignant fibrous histiocytoma (MFH) occurs most commonly in the extremities (70–75%, with lower extremities accounting for 59% of cases), followed by the retroperitoneum. Tumors typically arise in deep fascia or skeletal muscle. MFH has been reported to occur in the lung, kidney, bladder, scrotum, vas deferens, heart, aorta, stomach, small intestine, orbit, CNS, paraspinal area, dura mater, facial sinuses, nasal cavity, oral cavity, nasopharynx, and soft tissues of the neck. Although metastases to the lung are common, primary lung malignant fibrous histiocytoma is extremely rare. We report a case of a 54-year-old male who in 1995 was histologically diagnosed of having primary lung MFH, reviewing the clinical and radiographic findings. He had a 15-pack-year history of cigarette smoking but had not smoked since 1986. The patient underwent surgical tumor resection but didn't receive any adjuvant therapy. Since then multiple metastatic sites have developed but he is still alive.

## 2. Case Report

**Figure 1:**
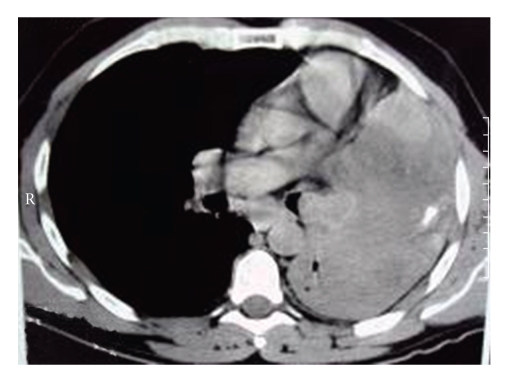
Tumor mass obstructing the left main bronchus.

The patient was admitted to our hospital for the first time in 1986 when he underwent a chest X-ray, as part of routine examination, which revealed a tumor shadow on left lung. Physical examination revealed decreased breath sounds over the left basal lung field. Complete blood count, serum biochemistry and tumor markers (including carcinoembryonic antigen, tissue plasmin antigen, and squamous cell carcinoma antigen), were within normal limits. He denied to undergo bronchoscopy and returned home. The patient was readmitted to our hospital in 25/4/1995 suffering from severe cough, mucous expectoration and shortness of breath over a three-month-period. Radiologic examinations included chest chest X-rays and chest computerized tomography (CT) scan. The spiral chest CT scan revealed a large tumor of soft tissue density replacing most of the left lung and occluding the lumen of the left main bronchus, associated with atelectasis (Figure 1). A technetium-99m methylene diphosphonate (ıTc-MDP) bone scan failed to show bone metastases.

**Figure 2:**
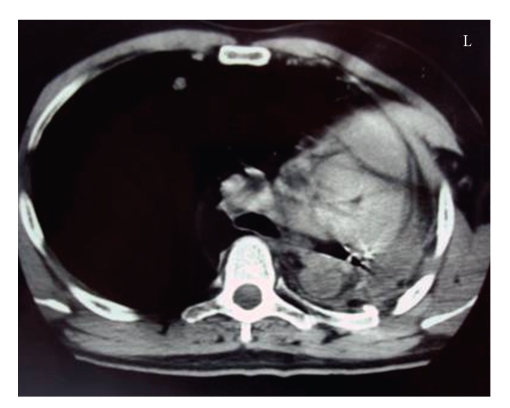
Left pneumectomy; Nodular matastases.

Under these circumstances, the patient was consented for surgery and a left pneumonectomy was performed (Figure 2). Histological studies of the mass revealed a poorly differentiated mesenchymal neoplasm, consistent with malignant fibrous histiocytoma. In addition, an immunohistochemical examination of the specimen showed positive staining for CD68, *α*1-antichymotrypsin and factor Xlla. No adjuvant chemotherapy or irradiation was given, and the patient went home after 18 days of hospitalization and follow up CT scans every six months.

In 1997, the patient presented a nodule on his left shoulder and underwent excision and biopsy. The histological examination demonstrated tumor of mesenchymal origin which presented similar characteristics with the resected lung tumor.

**Figure 5:**
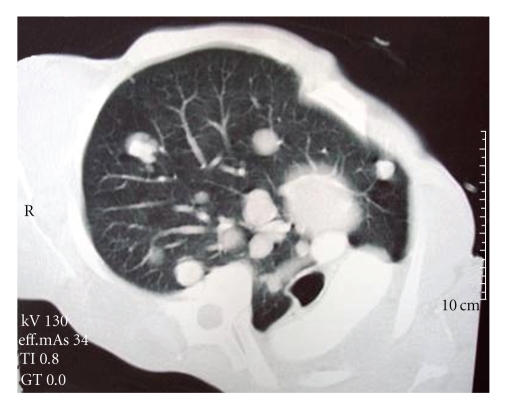
Innumerable metastatic nodules with involvement of thoracic wall.

In 2008, a chest CT scan showed innumerable, well defined of variable size hypodense nodules which presented inhomogeneous contrast enhancement and scattered in the right lung, the left postpneumonectomy space and the mediastinum. It also revealed lesions of soft tissue density in various parts of the thoracic wall, some of which were associated with rib destruction (Figure 5). Furthermore, the abdominal CT scan showed multiple circumscribed masses occupying the retroperitoneal space, which displaced the intestine anteriorly.

## 3. Discussion

MFH is the most common soft tissue sarcoma in adults, comprising about 10% of all sarcomas, usually arising in the extremities and trunk [1]. It may occur both in children and infants [2]. It is an aggressive tumor with high potential of local relapse and distant metastases. According to reported studies lung is the most common site for distant metastases [3].

We must take into consideration the fact that primary MFH of the lung is extremely rare (0,2% of the pulmonary neoplasms) with only about 50 cases been reported in the literature [4]. There must be no evidence of another primary site as determined by physical and radiologic exam before a MFH can be considered a lung primary tumor. In our case, no other primary site of disease was identified.

We must bear in mind that the majority of patients present chest pain, dyspnea, cough, and hemoptysis. Less common symptoms are hypertrophic pulmonary osteoarthropathy, hypoglycaemia, and neutrophilia. In addition, patients may be asymptomatic during their presentation.

On the other hand, primary lung MFH of the lung appears on CT as a peripheral tumor of soft tissue density sometimes with areas of low attenuation centrally, bud rarely cavitated. Despite the fact that has been found pleural effusion in 20% of patients calcification has been described in only one adult patient in the literature [5]. Although the radiologic manifestations have no differential points from other lung tumors, chest CT to define of the size and location of the tumor and the extent of the adjacent tissues involvement.

Besides, the final diagnosis is based on microscopic and immunohistochemical studies [6], histologic typing is based on Enzinger and Weiss classification [7] (pleomorph/storiform, myxoid, giant-cell, and inflammatory types). Furthermore, the treatment of choice for primary lung MFH is a complete surgical resection with negative microscopic margins, associated to improved disease free specific survival [8]. Metastases may appear months or years after resection of the primary lesion.

Halyard et al. describe in their review eight patients with primary lung MFH alive without evidence of disease more than 5 years after the diagnosis (two of them for 10 years) and one patient who died of the disease after a 6-year period. Regnar et al. reported two patients who survived 11 years after the operation [9]. Rzyman et al. reported one patient alive with no evidence of disease 11 years postoperatively.

Moreover, survival after complete surgical resection in cases of primary lung MFH has been reported to be better than for other pulmonary sarcomas [4]. Many clinicopathologic factors are significally related to disease free specific survival (DSS) and metastasis free survival (MFS), including age, tumor size-depth, classification stage, histologic type and grade. As with other forms of soft tissue sarcomas histopathologic grade is the most important factor determinant in the characterization of disease stage [8]. In the current case, most of these factors were associated with adverse outcome, so we assume that the long patient's standing survival is related to a low grade primary tumor.

Last but not least, the role of adjuvant radiotherapy and chemotherapy has not definitely been defined yet. Le Doussal et al. and Mills et al. clearly concluded that radiotherapy significantly reduces the risk of local relapse. Edbronson et al reported no improvement after systematic chemotherapy [10], but again Le Doussal et al. proposed adjuvant chemotherapy in patients at increased risk for distant metastasis.

## 4. Conclusion

To put it in a nutshell, we must take into account that primary sarcomas of the lung are rare. Patients with MFH of the lung must be carefully evaluated to rule out a metastatic origin, as MFH is primarily a soft tissue tumor of the extremities. Rates of local and distant recurrence remain high even after radical surgery, but rare cases of long-term survival have been reported. Histologic grade seems to be the most important prognostic factor for disease specific survival. Although the association between metastases and cancer-related deaths is strongly significant, our patient has survived over 14 years developing multiple metastases.

## Figures and Tables

**Figure 3:**
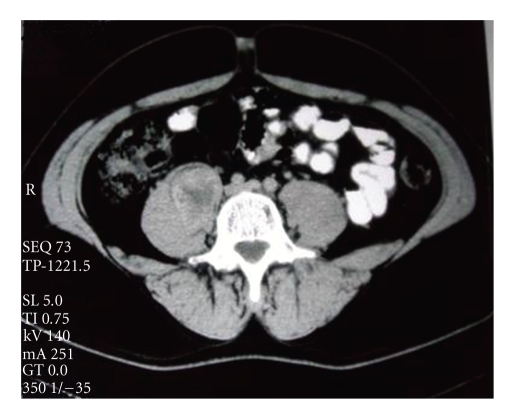
Metastatic lesion of the right iliopsoas muscle.

**Figure 4:**
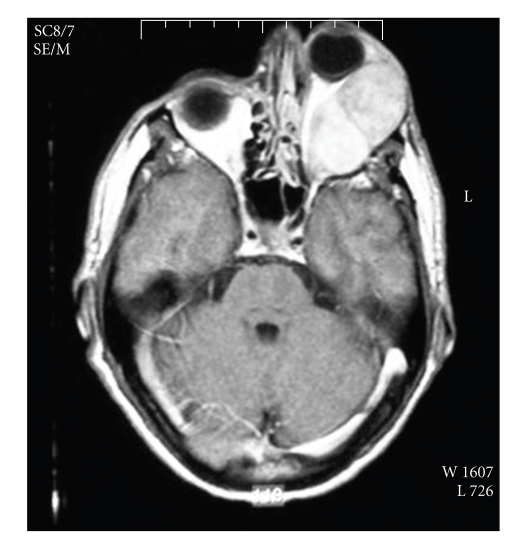
Left intraocular mass.

**Table 1:** 

12/1995Chest CT scan	Few nodular densities in the right lung
04/1996Abdominal CT scan	A metastasis to the right iliopsoas musle Figure 3
11/1997Chest CT scan	More nodules in the right lung with the largest being approximately 3 cm.
04/2004- Brain MRI	Intraoccular mass on the left site Figure 4
